# Non-antibiotic Small-Molecule Regulation of DHFR-Based Destabilizing Domains *In Vivo*

**DOI:** 10.1016/j.omtm.2019.08.002

**Published:** 2019-08-15

**Authors:** Hui Peng, Viet Q. Chau, Wanida Phetsang, Rebecca M. Sebastian, M. Rhia L. Stone, Shyamtanu Datta, Marian Renwick, Yusuf T. Tamer, Erdal Toprak, Andrew Y. Koh, Mark A.T. Blaskovich, John D. Hulleman

**Affiliations:** 1 Department of Ophthalmology, University of Texas Southwestern Medical Center, 5323 Harry Hines Blvd., Dallas, TX 75390, USA; 2 Centre for Superbug Solutions, Institute for Molecular Bioscience, The University of Queensland, 306 Carmody Road, Brisbane, QLD 4072, Australia; 3 Department of Chemistry, Massachusetts Institute of Technology, 77 Massachusetts Ave., Cambridge, MA 02139, USA; 4 Green Center for Systems Biology, University of Texas Southwestern Medical Center, 5323 Harry Hines Blvd., Dallas, TX 75390, USA; 5 Department of Pharmacology, University of Texas Southwestern Medical Center, 5323 Harry Hines Blvd., Dallas, TX 75390, USA; 6 Department of Pediatrics, University of Texas Southwestern Medical Center, 5323 Harry Hines Blvd., Dallas, TX 75390, USA; 7 Department of Microbiology, University of Texas Southwestern Medical Center, 5323 Harry Hines Blvd., Dallas, TX 75390, USA

**Keywords:** chemical biology, trimethoprim, non-antibiotic, gene therapy, conditional regulation, destabilizing domain, ocular, hepatic

## Abstract

The *E. coli* dihydrofolate reductase (DHFR) destabilizing domain (DD), which shows promise as a biologic tool and potential gene therapy approach, can be utilized to achieve spatial and temporal control of protein abundance *in vivo* simply by administration of its stabilizing ligand, the routinely prescribed antibiotic trimethoprim (TMP). However, chronic TMP use drives development of antibiotic resistance (increasing likelihood of subsequent infections) and disrupts the gut microbiota (linked to autoimmune and neurodegenerative diseases), tempering translational excitement of this approach in model systems and for treating human diseases. Herein, we identified a TMP-based, non-antibiotic small molecule, termed 14a (MCC8529), and tested its ability to control multiple DHFR-based reporters and signaling proteins. We found that 14a is non-toxic and can effectively stabilize DHFR DDs expressed in mammalian cells. Furthermore, 14a crosses the blood-retinal barrier and stabilizes DHFR DDs expressed in the mouse eye with kinetics comparable to that of TMP (≤6 h). Surprisingly, 14a stabilized a DHFR DD in the liver significantly better than TMP did, while having no effect on the mouse gut microbiota. Our results suggest that alternative small-molecule DHFR DD stabilizers (such as 14a) may be ideal substitutes for TMP in instances when conditional, non-antibiotic control of protein abundance is desired in the eye and beyond.

## Graphical Abstract

**Figure:**
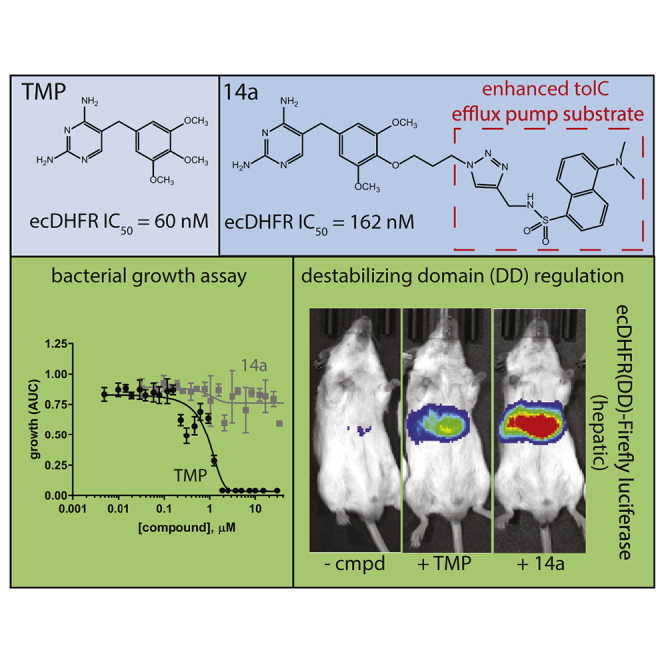

## Introduction

Gene therapy aims to modify pathological phenotypes and provide disease treatment by the introduction of transgenes via recombinant viral vectors (e.g., recombinant adeno-associated virus [rAAV] or lentivirus) or non-viral vectors (naked DNA, nanoparticles, etc.).^1^ While gene therapy for loss-of-function diseases show promise,^2^ strategies implementing regulatable gene expression are ideal for avoiding potential toxicity or unwanted effects of overexpression^3^ by enabling researchers with the means of turning “on” transgenes to induce appropriate degrees of expression when necessary and turning “off” when unnecessary or detrimental. Conventional regulatable systems such as “Tet-ON” and “Tet-OFF”^4^ regulate transgene expression at the transcriptional level and require days to weeks for full activation and deactivation.^5^, ^6^, ^7^, ^8^ Controlling expression directly at the protein level, such as through the use of destabilizing domains (DDs),^9^, ^10^ eliminates DNA to mRNA to protein processing time and allows for quicker regulation of protein abundance.

DDs are genetically engineered domains that are inherently unstable and rapidly ubiquitinated and degraded by the proteasome, unless the DD is stabilized by a small-molecule pharmacologic chaperone.^9^ Use of the *Escherichia coli* (*E. coli*) dihydrofolate reductase (DHFR) DD is appealing, due to its stabilizing ligand, trimethoprim (TMP), an inexpensive and well-characterized compound that can cross both the blood-brain barrier (BBB)^10^ and the blood-retinal barrier (BRB)^11^ and that is highly specific for *E. coli* DHFR.^12^ In the presence of TMP, fusion proteins containing DHFR DDs are readily expressed and resistant to proteasomal degradation, thus allowing for positive regulation.^10^ The DHFR DD system is also reversible in that TMP can be washed out *in vitro* and metabolized or excreted *in vivo*. This generalized model system has been confirmed to be effective in controlling the abundance of numerous fusion proteins^13^, ^14^, ^15^, ^16^, ^17^, ^18^, ^19^ in several tissues, including the brain^10^, ^20^, ^21^ and the eye,^11^, ^22^ in a spatial, temporal, and dose-dependent manner.

TMP is a commonly used first-line antibiotic for treating urinary tract infections^23^ and pneumonia,^24^ among other infections,^25^ due to its ability to inhibit *E. coli* DHFR. Being commercially available and inexpensive, TMP is a good candidate for use in gene therapy applications in conjunction with the DHFR DD.^10^ Unfortunately, the antibiotic properties of TMP make it less alluring for long-term and repeated utilization in the application of the DHFR DD system, which requires frequent administration of the small-molecule chaperone whenever positive gene regulation is desired. Chronic antibiotic use is associated with the rise of antibiotic-resistant bacteria and infections,^26^, ^27^ leading to the estimated worldwide deaths of at least 700,000 people per year, a figure that has been estimated to increase to 10 million annually by 2050 (https://amr-review.org/). Frequent usage of TMP by itself, or combined synergistically with sulfamethoxazole (SMX), as is normally prescribed for infections, has led to the development of resistance in many bacterial strains, primarily through point mutations in the *E. coli* DHFR promoter region or coding sequence.^28^, ^29^, ^30^ Additionally, TMP-induced selective pressure has been demonstrated to cause mutations to multidrug-resistance genes,^31^ which can render bacteria resistant not only to TMP but also to multiple diverse classes of antibiotics^32^ and which are a significant threat to public health (https://www.cdc.gov). Even sub-minimal inhibitory concentrations of antibiotics can lead to high levels of antibiotic resistance.^33^ Therefore, unnecessary antibiotic use other than treating related infections, such as using TMP for regulating gene therapies, should be limited in order to reduce the risk of antibiotic resistance. Moreover, the oral administration of antibiotics often leads to disruption of the gut microbiota, which is a complex micro-ecosystem that can affect human physiology, cause inflammatory and neurodegenerative diseases, and even play a role in ocular disease pathogenesis (e.g., uveitis^34^ and age-related macular degeneration [AMD])^35^ by contributing metabolic resources and immune factors.^36^, ^37^, ^38^, ^39^, ^40^ In fact, harsh antibiotics, like clindamycin, can alter the gut bacterial community composition as soon as 24 h post-administration and require as long as 2 years for complete restoration to the original bacterial composition.^38^, ^41^, ^42^, ^43^

Our group has previously established the proof of concept of stabilizing DHFR DDs using TMP in the mouse eye.^11^ However, due to the potential for adverse effects after long-term antibiotic usage, we sought to identify a surrogate molecule for TMP that can stabilize DHFR DDs but without conferring antibiotic properties or having significant effects on the gut microbiota. In this study, we verified that 14a, a TMP-derived compound, can substitute for TMP to stabilize DHFR DDs both *in vitro* and *in vivo*. In contrast to TMP, which, we show, induces alterations in mouse gut microbiota, 14a does not inhibit bacterial growth and has no significant impact on mouse gut flora. Overall, the absence of antibiotic properties of 14a, along with its comparable efficacy to that of TMP, bolsters the feasibility of using a non-antibiotic compound with DHFR DDs as a strategy for regulation of protein abundance in the eye, liver, and elsewhere.

## Results

### A Single Dose of TMP Induces Alterations of Gut Microbiota in Mice

**Figure 1:**
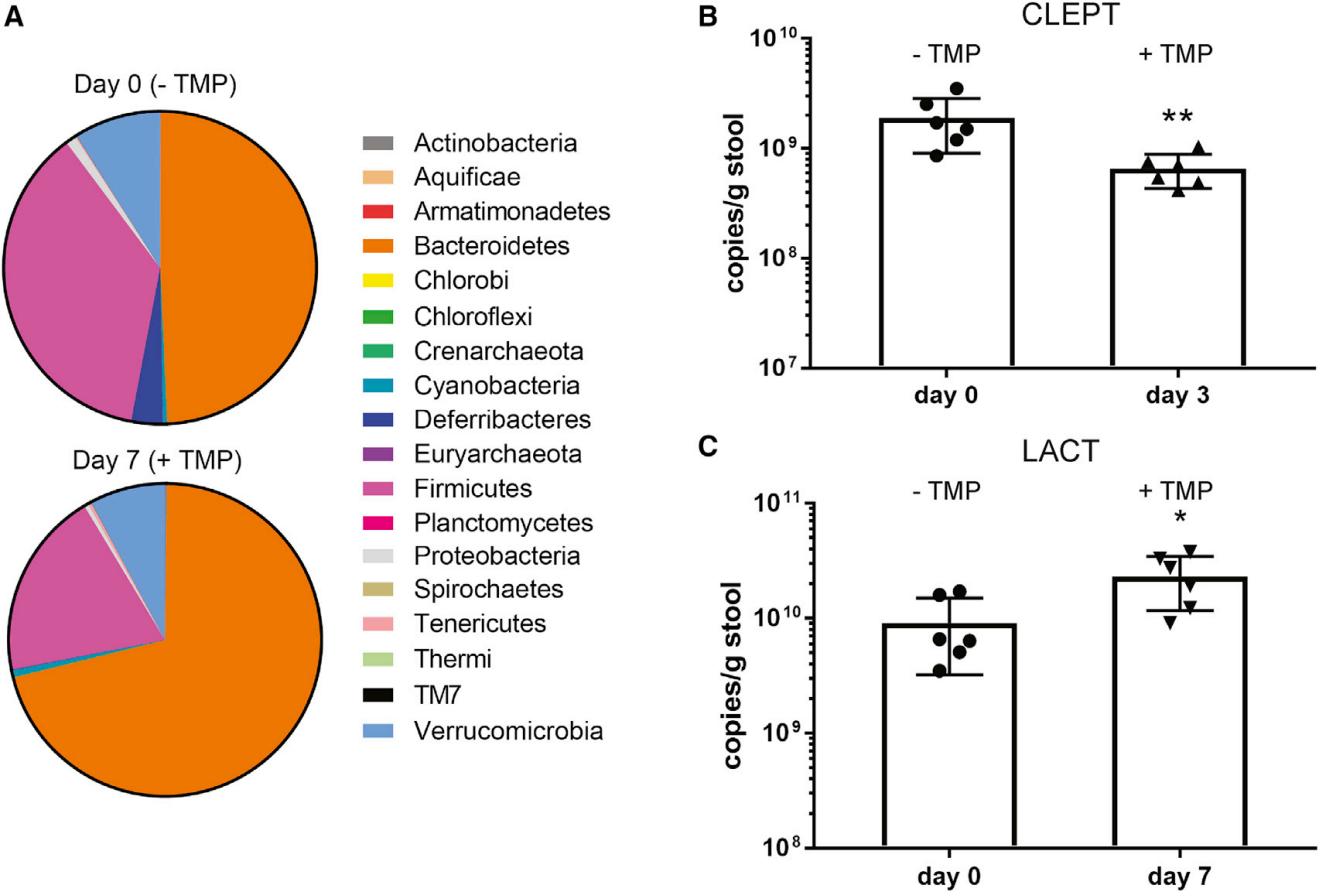
Disruption of Mouse Gut Microbiota by a Single-Dose Treatment of TMP (A) Pie charts showing the percentage of each phylum detected in mouse fecal samples before treatment (day 0) and 7 days after TMP treatment (1 mg per mouse via gavage). The mean values of n = 6 mice are shown. (B and C) Quantitation of the abundance of the (B) CLEPT group and (C) LACT group in mouse feces before (day 0) and after (day 3 or 7) TMP treatment. Data are represented as mean ± SD of n = 6; statistical analysis by Mann-Whitney test. *p < 0.05; **p < 0.01.

It is now well known that maintaining a balanced gut microbiota is critical for human health, and alterations in gut flora are often observed in mammals after the administration of antibiotics.^41^, ^44^ However, little is known about the effects of only TMP administration, which is commonly used in combination with SMX, on the gut microbiota. So far, only two studies have reported that TMP-SMX treatment substantially changes human gut microbiota,^45^, ^46^ yet no published research, to our knowledge, has studied the impact of only TMP. For this reason, we conducted comprehensive 16S rRNA sequencing to analyze the effects of TMP on the relative levels of bacteria in the mouse gut microbiota. We treated the mice with a single low dose of 1 mg TMP by oral gavage (50 mg/kg in the mouse, a human equivalent dose of 4.07 mg/kg,^47^ which is considered “low-dose” TMP^48^) to mimic one antibiotic dosage, although in practicality, regulation of the DHFR DD system in a gene therapy context would consist of long-term and frequent treatment using TMP. We collected fecal samples at day 0 (prior to treatment), day 3, and day 7 and extracted genomic DNA for sequencing. We found that, among the major phyla, the proportion of Firmicutes in total bacteria decreased substantially by ∼2-fold from 37% to 19%, and Bacteroidetes levels increased markedly from 49% to 71% between day 0 and day 7 (Figure 1A). In addition, we performed qPCR experiments on mouse fecal samples collected at day 0, day 3, and day 7 to quantify the abundance of total bacteria (eubacteria; EUB) and several representative gut bacteria, including Bacteroides (BACT), Enterobacteriaceae (ENTERO), the *Eubacterium rectale*/*Clostridium coccoides* (EREC) group, the *Clostridium leptum* (CLEPT) group, and the *Lactobacillus*/*Enterococcus* (LACT) group. We observed that CLEPT group levels are significantly decreased at day 3 (Figure 1B) and that the abundance of the *Lactobacillus*/*Lactococcus* group increased significantly at day 7 (Figure 1C) after a single administration of TMP. The amount of total bacteria (EUB) and other species (BACT, ENTERO, and EREC) quantified remained similar after TMP treatment (Figures S1A–S1F). While we found significant changes in the representative gut bacteria after TMP treatment, it is unclear whether or how these changes would ultimately culminate in affecting mouse biology. Nonetheless, these changes are a result of a single TMP treatment, which is an unlikely regime for regulating DHFR DD abundance. The observed effects of more frequent TMP administration (i.e., daily or weekly) on the gut microbiota and antibiotic resistance would be expected to be increasingly significant and concerning.

### The TMP Derivative, 14a, Has No Significant Impact on Bacterial Growth and Mouse Gut Microbiota

**Figure 2:**
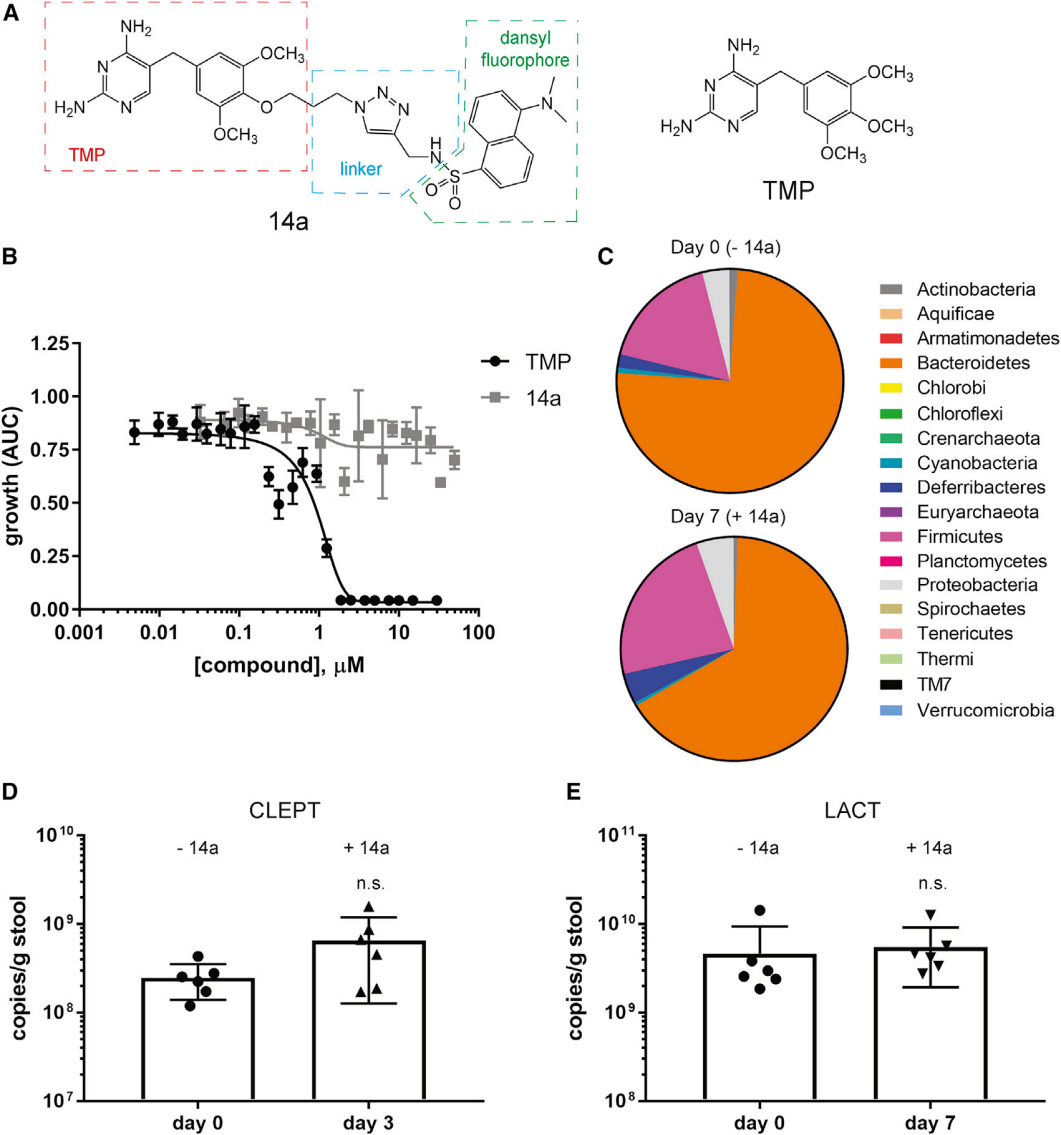
Minimal Impact of 14a on Bacterial Growth and Mouse Gut Microbiota (A) Chemical structures of 14a and TMP. (B) Endpoint turbidity (OD_600_) of *E. coli* bacterial culture after 19–24 h of treatment with different concentrations of TMP or 14a. (C) Pie charts showing the percentage of each phylum detected in mouse feces samples before (day 0) and at day 7 after 14a treatment (3 mg per mouse via gavage). The mean values of n = 5 mice are shown. (D and E) Quantitation of the abundance of the (D) CLEPT and (E) LACT groups in mouse fecal samples before (day 0) and after (day 3 or 7) 14a treatment. Data are represented as mean ± SD of n = 6; statistical analysis by Mann-Whitney test. n.s., not significant.

Due to the disruption of the mouse gut microbiota by TMP, even after a single dose, we next searched the literature for non-antibiotic TMP substitutes that would theoretically allow for stabilization of DHFR DDs without disrupting the microbiome. Phetsang and colleagues conjugated fluorescent moieties on to position 4 of the TMP phenyl ring in an effort to follow the subcellular localization of TMP inside bacteria.^49^ Select compounds (12a, 12b, 13a, and 14a; Figure 2A; Figure S2A) were able to retain *E. coli* DHFR half-maximal inhibitory concentration (IC_50_) values (148–254 nM) similar to that of TMP (60 nM) in biochemical assays, which indicated an ability of the compounds to bind to *E. coli* DHFR.^49^ However, surprisingly, these compounds were unable to prevent bacterial growth at the highest concentration used (minimal inhibitory concentration [MIC] ≥ 64 mg/mL), due to enhanced efflux from the bacteria via the TolC-dependent efflux pump.^49^ We realized that these TMP derivatives may serve as ideal candidates for stabilizing DHFR DDs,^11^, ^15^, ^50^ since they retain the ability to bind to *E. coli* DHFR while minimizing the side effects of administering an antibiotic *in vivo*.

First, we verified that a representative molecule from this series, 14a, did not possess antibiotic activity. BW25113 wild-type (WT) *E. coli* were treated with increasing concentrations of TMP or 14a, and bacterial growth was monitored for up to 24 h. In contrast to TMP, which completely inhibited *E. coli* growth at ∼1.9 μM, concentrations as high as 50 μM 14a had no impact on bacterial growth (Figure 2B). To further confirm the non-antibiotic characteristics of 14a, similarly to TMP, we treated mice with a single dose of 3 mg 14a (a molar equivalent to TMP); collected feces samples at day 0 (prior to treatment), day 3, and day 7; extracted genomic DNA from mouse fecal samples; and analyzed them by 16S rRNA sequencing and qPCR. The two major phyla, Firmicutes and Bacteroidetes, which were substantially altered in TMP samples (Figure 1B), were minimally changed from 17% to 23% and reduced from 75% to 66%, respectively (Figure 2C). To parallel our analysis performed with TMP-treated mice, we also quantified the abundance of total bacteria and several representative gut bacteria in fecal samples from mice treated with 14a by qPCR. As expected, there were no significant changes in the levels of any of the bacteria tested after 14a treatment (Figures 2D and 2E; Figures S3A–S3F).

### Stabilization of DHFR DDs by 14a in Mammalian Cells

Next, we examined whether the non-antibiotic TMP-derivative compounds could stabilize DHFR DDs, as suggested by their IC_50_ values for *E. coli* DHFR inhibition.^49^ We screened 12a, 12b, 13a, and 14a in HEK293A cells transfected with a destabilized yellow fluorescent protein (DHFR.YFP) and found that each of the compounds could dose-dependently stabilize DHFR.YFP (Figures S2B and S2C). The stabilization ability of the compounds was similar among 12a, 12b, and 14a but weaker with 13a with respect to fold induction of DHFR.YFP (Figures S2B and S2C). Thus, we prioritized 14a for subsequent characterization, since both 12a and 12b contain a reactive nitro (NO_2_) group that is contraindicated by most medicinal chemists due to mutagenicity and genotoxicity (Figure S2A)^51^ and because 14a demonstrated an enhanced ability to stabilize DHFR.YFP compared to 13a (Figure S2C). Importantly, 14a retained the selectivity for *E. coli* DHFR, as recombinant human DHFR has similar specific activity when treated with TMP versus 14a (Figures S4A and S4B).

**Figure 3:**
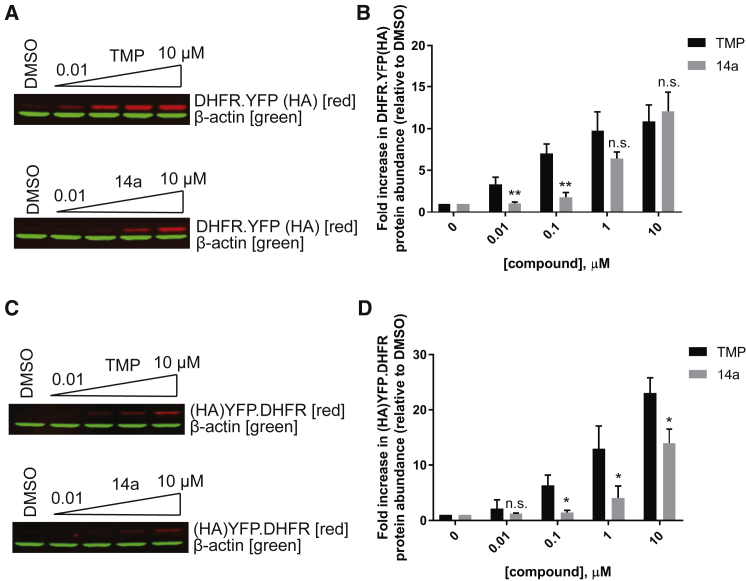
Stabilization of DHFR DD by 14a in HEK293A Cells (A and C) Western blot probing (A) DHFR.YFP or (C) YFP.DHFR expression using the HA tag antibody at different doses of vehicle, TMP, or 14a after 24 h of treatment. β-actin was used as an internal control. Representative image of three experiments. (B and D) LI-COR quantitation of (B) DHFR.YFP or (D) YFP.DHFR band intensity relative to DMSO-treated sample. Data are represented as mean ± SD of n = 3; statistical analysis by unpaired, two-tailed t test assuming equal variance compared to samples treated with the same concentration of TMP. *p < 0.05; **p < 0.01; n.s., not significant.

We next compared the ability of 14a to stabilize a DHFR DD to the canonical ligand, TMP. HEK293A cells were transfected with YFP fused to either an N-terminal DHFR (DHFR.YFP) or a C-terminal DHFR (YFP.DHFR)^10^ and then treated in parallel with increasing doses of 14a or TMP, and DHFR.YFP and YFP.DHFR abundance was assessed by western blot. We found that both 14a and TMP stabilized N-terminal and C-terminal DHFR DDs in a dose-dependent manner (Figures 3A–3D). 14a could stabilize N-terminal DHFR DDs (R12Y/G67S/Y100I variant) as well as TMP at high doses (≥1 μM) but not as well at low concentrations (≤0.1 μM) (Figures 3A and 3B), as might be expected from its ∼3-fold less potent *E. coli* DHFR IC_50_. The ability of 14a to stabilize C-terminal DHFR DDs (N18T/A19V variant) was not as effective as that of TMP, but the fold increase induced by 14a at 10 μM was similar to N-terminal DHFR stabilization by 14a (∼12- to 14-fold) (Figures 3B and 3D).

**Figure 4:**
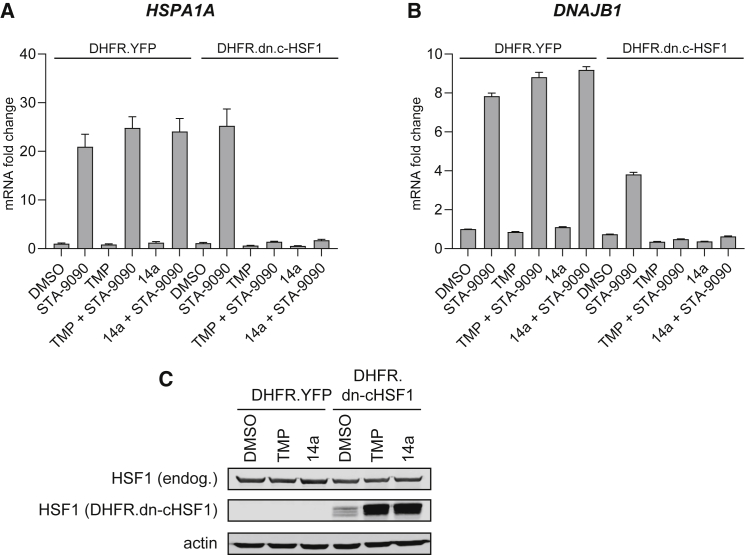
Regulation of HSF1 Target Genes by 14a-Stabilized DHFR.dn-cHSF1 in HEK293T-Rex Cells (A and B) The expression levels of (A) HSPA1A and (B) DNAJB1 under different treatment conditions, normalized to vehicle (DMSO)-treated HEK293T-Rex control cells stably expressing DHFR.YFP. Data presented are the mean ± SD of technical triplicates. (C) Western blot probing endogenous HSF1 and DHFR.dn-cHSF1 in cells treated with DMSO, 10 μM TMP, or 14a for 24 h. β-actin was used as the internal control. Representative image of three independent experiments.

To verify that the stabilization of DHFR DDs by 14a was not specific to the fusion protein, YFP, and that 14a could be used more broadly to control cellular signaling pathways, we next tested 14a stabilization of a dominant-negative, constitutively active heat shock factor 1 (dn-cHSF1) fused to an N-terminal DHFR (DHFR.dn-cHSF1).^52^ Stabilization of DHFR.dn-cHSF1 should, consequently, repress the expression of the HSF1 target genes *HSPA1A* and *DNAJB1*, both basally and upon heat shock response activation by STA-9090, an HSP90 inhibitor and HSF1 activator.^53^ As expected, stable HEK293T-Rex cells expressing DHFR.YFP demonstrated induction of *HSPA1A* and *DNAJB1* upon STA-9090 treatment by 20.9-fold and 7.8-fold, respectively, which was unaffected by 14a or TMP treatment (Figures 4A and 4B). STA-9090 similarly upregulated these genes in DMSO-treated DHFR.dn-cHSF1 stable cells as well by 25.3-fold and 3.8-fold, respectively, but failed to do so when the cells were pre-treated with 10 μM 14a or TMP (Figures 4A and 4B). These data suggest that both 14a and TMP can effectively and similarly stabilize DHFR.dn-cHSF1 (confirmed by western blot; Figure 4C) and conditionally repress HSF1 signaling.

**Figure 5:**
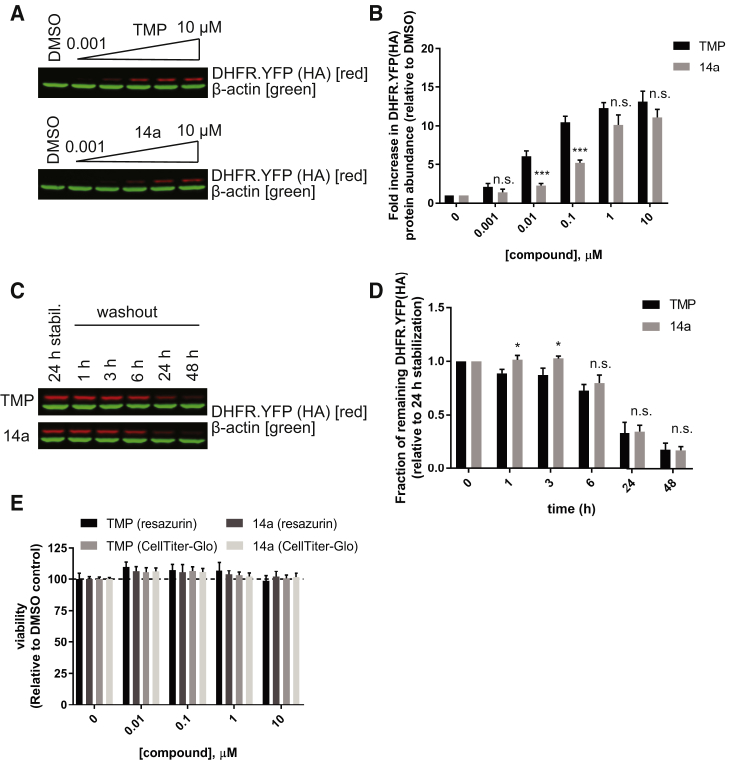
Stabilization and Washout of DHFR.YFP by 14a in ARPE-19 Cells (A) Western blot probing DHFR.YFP (using the HA tag antibody) at different doses of TMP and 14a or vehicle reagent, DMSO, 24 h after treatment. (B) LI-COR quantitation of DHFR.YFP band intensity relative to DMSO-treated cells. (C) Western blot probing DHFR.YFP (using the HA tag antibody) over a 48-h time course of washout after an initial 24 h of stabilization by 10 μM TMP or 14a. (D) LI-COR quantitation of DHFR.YFP band intensity relative to the levels of 24 h of stabilization. β-actin was used as an internal control. Representative image of three independent experiments. Data are presented as mean ± SD of n = 3; statistical analysis by unpaired, two-tailed t test assuming equal variance compared to TMP-treated samples under the same condition. *p < 0.05; ***p < 0.001; n.s., not significant. (E) The viability of ARPE-19 cells is unaffected by TMP or 14a. Numbers are normalized to vehicle-treated (DMSO) cells. Data are presented as mean ± SD of n = 3. No significant differences were observed between TMP- and 14a-treated cells or among different doses of compound.

Since our research focus is gene therapy for eye diseases, we further tested 14a in an ocular-derived cell line, ARPE-19. We conducted a parallel treatment of ARPE-19 cells expressing DHFR.YFP with different doses of TMP or 14a. Comparably, 14a showed nearly the same fold induction in DHFR.YFP as TMP at high doses (≥1 μM) but was not as effective at lower concentrations (≤0.1 μM) (Figures 5A and 5B). Reversibility is an important property of the DHFR DD system, so we next examined the kinetics of washout of 14a, in parallel with TMP, after 24 h stabilization with 10 μM compound. 14a presented nearly identical kinetics of washout as TMP (Figures 5C and 5D). Finally, before using 14a *in vivo*, we evaluated the cytotoxicity of 14a and compared it to that of TMP in ARPE-19 cells by two different viability assays; the resazurin cell viability assay (mitochondrial reduction potential) and the CellTiter-Glo assay (ATP levels). Both 14a and TMP exhibited no cytotoxicity in ARPE-19 cells in either of these assays (Figure 5E).

### 14a Is Able to Stabilize DHFR DDs in the Retina and the Liver of Mice

**Figure 6:**
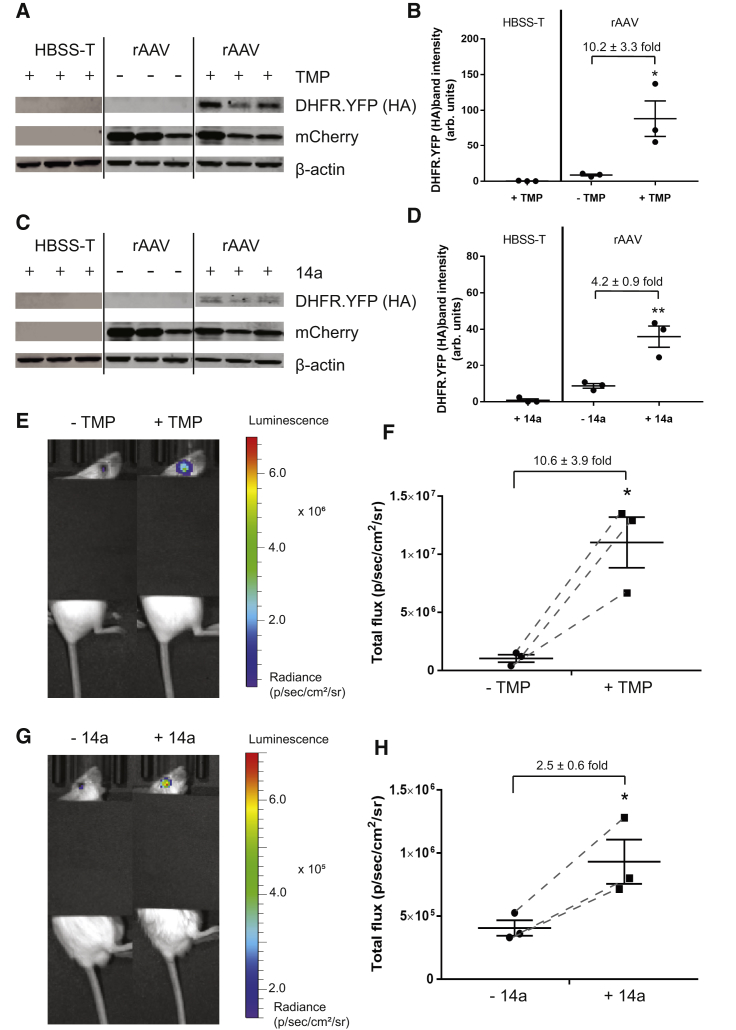
Stabilization of DHFR DDs by TMP and 14a in the Retina of Mice (A and C) Expression of DHFR.YFP (using the HA tag antibody) and mCherry were detected by western blot in mouse neural retina tissue 6 h after (A) TMP treatment or (C) 14a oral gavage. Triplicate data of independent mice are shown. Note: (A) and (C) use the same untreated, injected (rAAV, no TMP or 14a) mice as a control. (B and D) LI-COR quantitation of DHFR.YFP band intensity in (A) TMP or (C) 14a treated samples. Data are presented as mean ± SEM of n = 3; statistical analysis by unpaired, two-tailed t test assuming equal variance compared to rAAV-injected “−TMP” or “−14a” samples. *p < 0.05; **p < 0.01. (E and G) Bioluminescence signals originating from a representative mouse were obtained before (−TMP or −14a) and 6 h after (E) TMP treatment (1 mg per mouse via gavage; +TMP) or (G) 14a treatment (3 mg per mouse via gavage; +14a). Representative images of three individual mice. (F and H) Quantification of bioluminescence imaging. The total flux numbers of n = 3 mice are plotted before and after (F) TMP treatment or (H) 14a treatment. Dashed lines indicate the change before and after treatment of the same mouse. Data are represented as mean ± SEM; statistical analysis by unpaired, two-tailed t test assuming equal variance compared to the data before TMP or 14a treatment. *p < 0.05.

Ultimately, we see that the true utility of the DHFR DD system lies in translating its use as a tool for probing biology into *in vivo* application as a potential gene therapy strategy for controlling stress-responsive signaling pathways. As such, we next validated the 14a-regulated DHFR DD system *in vivo* in the retina of mice. The eye is an ideal organ for testing gene therapies due to its accessibility, immune-privilege, and transparency; however, it is a challenging system with respect to drug delivery of molecules originating systemically due to the BRB. C57BL/6J mice intravitreally injected with rAAV expressing DHFR.YFP and a concomitantly expressed mCherry were given an equal molar amount of TMP or 14a (1 mg and 3 mg, respectively) by oral gavage. After 6 h, the mice were sacrificed, and their retinas were homogenized and probed for DHFR.YFP by western blot. DHFR.YFP abundance was significantly elevated in TMP- and 14a-treated mice, indicating that 14a can successfully cross the BRB and stabilize DHFR.YFP in the retina, although the induction fold increase of DHFR.YFP by 14a was lower than that induced by TMP (4.2-fold versus 10.2-fold) (Figures 6A–6D). In separate experiments, we also verified the ability of 14a to stabilize a sensitive and quantitative luciferase reporter, firefly luciferase (DHFR.FLuc), in the eyes of live mice. Non-pigmented BALB/c mice were intravitreally injected with rAAV encoding DHFR.FLuc and were allowed to express the protein over 10 days. Six hours post-gavage of 1 mg TMP or 3 mg 14a, live mice were imaged using bioluminescence, and the resulting signal was compared to the luciferase signal prior to compound administration. We observed that the levels of bioluminescence signal significantly increased by 2.5-fold at 6 h after 14a treatment, suggesting that 14a is able to stabilize DHFR DDs in the retina of mice, though this fold increase was not as effective as TMP-based induction (10.6-fold, Figures 6E–6H).

**Figure 7:**
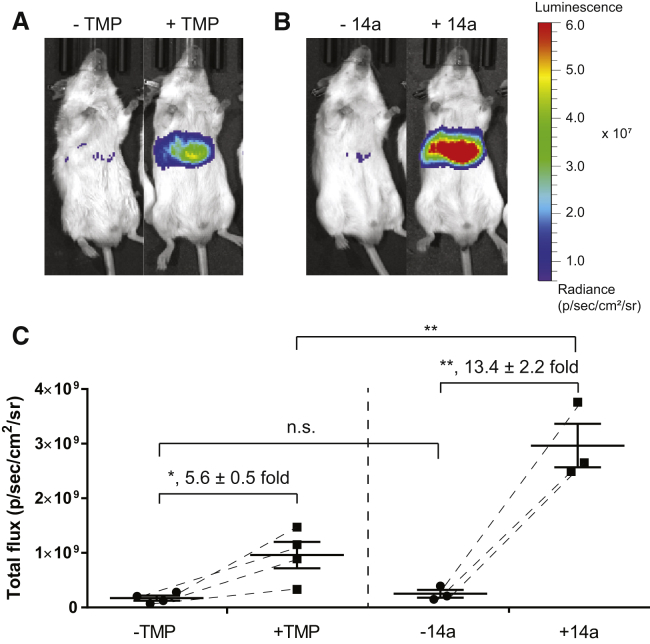
Stabilization of DHFR.FLuc by TMP and 14a in the Liver of Mice (A and B) Bioluminescence image of a representative mouse obtained before (−TMP or −14a) and 6 h after TMP treatment (1 mg per mouse via gavage; +TMP) (A) or 14a treatment (3 mg per mouse via gavage; +14a) (B). Representative images of three (14a treatment) or four (TMP treatment) individual mice. (C) Quantification of bioluminescence imaging. The total flux numbers of n = 4 mice are plotted before and after TMP or n = 3 mice of 14a treatment. Dashed lines indicate the change before and after treatment of the same mouse. Data are represented as mean ± SEM; statistical analysis by unpaired, two-tailed t test assuming equal variance. *p < 0.05; **p < 0.01; n.s., not significant.

To explore whether 14a could also be utilized to control DHFR DDs in organs other than the eye, we expressed DHFR.FLuc using rAAV in the liver of BALB/c mice for 2 weeks. Baseline FLuc luminescence in untreated mice was measured the day prior to administration of 1 mg TMP or 3 mg 14a by oral gavage. Six hours post-gavage, live mice were imaged using bioluminescence, and the resulting signal was compared to the baseline signal taken the day before. Both TMP and 14a stabilized DHFR.FLuc in the liver. However, in contrast to the regulation of a DHFR DD in the retina, 14a-mediated fold induction of FLuc signal in the liver was significantly higher than TMP-mediated fold induction (13.4 fold versus 5.6 fold, respectively, Figures 7A–7C), demonstrating that 14a is more effective in regulating DHFR DDs in the liver of mice. In conclusion, our *in vivo* studies indicate that TMP stabilizes DHFR DDs better in the retina than in the liver, while in contrast, 14a worked more effectively in the liver than in the retina. These observations may be due to differences in pharmacokinetics, pharmacodynamics, tissue penetration, and/or routes of excretion and/or metabolism between the two compounds; nonetheless, they indicate that both molecules can be used throughout the body for conditional protein stabilization.

## Discussion

In this study, we have established that 14a, a non-antibiotic TMP derivative that is an enhanced substrate for bacterial TolC-dependent efflux pumps, preserves the ability of TMP to stabilize DHFR DDs both *in vitro* and *in vivo* and has virtually no impact on the mouse gut microbiota (in contrast to TMP). Our study presents a significant optimization of the DHFR DD system and identifies the first demonstrated alternative stabilizer for such domains. It is intriguing to speculate that 14a or custom-made non-antibiotic DHFR DD stabilizers built on the 14a scaffold could be used in the future as a surrogate for TMP as a pharmacologic chaperone. We envision that our contributions will allow for safer application of this powerful protein abundance regulation method as a true gene therapy approach.

The constant use of TMP as an antibiotic has the potential to cause adverse physiologic effects. Oral administration of antibiotics, including TMP, can certainly cause alterations in gut microbiota, the effect of which may be long lasting. For example, one study indicated that healthy volunteers treated with antibiotics for 1 week or less experienced changes to their microbiota that persisted up to 2 years after treatment, with drastic loss of flora diversity and increased abundance of antibiotic-resistant bacterial strains and genes.^43^ Besides the well-known inflammatory and neurodegenerative disorders, such as Parkinson’s disease and Alzheimer’s disease, which can be influenced by the disruption of gut microbiota,^54^, ^55^ multiple connections have also been made recently that link the gut microbiota and ocular diseases. For example, gut microbiota is associated with autoimmune uveitis by regulating the levels of immune factors that migrate and infiltrate into the eye eventually.^56^, ^57^ In another study, microbial co-metabolites, particularly serotonin, derived from a low-glycemia diet were shown to be protective against AMD features.^35^ Whether such a drastic change would be observed in humans with only TMP administration is doubtful (since TMP is only bacteriostatic, not bactericidal) but, nonetheless, is a potential significant concern during a gene therapy scenario, since TMP would need to be consistently administered for long durations of time (years). While it is difficult to predict the ultimate biologic effects of disruption of the gut microbiota, it is reasonable to assert that minimizing alterations in the normal distribution and/or abundance of bacteria in the gut is probably an ideal strategy going forward. It is theoretically possible that there exists a concentration of TMP that can be administered systemically to control protein abundance but not act as an antibiotic in the gut, but this actual dose of TMP would rely on (1) where in the body stabilization of the DHFR DD is required; (2) the extent of the required stabilization (i.e., amplitude of stabilization); and (3) the time course of the desired treatment (i.e., the number of doses needed, e.g., once versus multiple times). Thus, it is nearly impossible to identify a single “one-size-fits-all” dose of TMP that would fulfill these criteria. Our identification and application of 14a circumvents these potential concerns.

Another concern of repeated use of an antibiotic is the exacerbation of antibiotic resistance. There is no doubt that antibiotic misuse or overuse, such as the use of TMP during scenarios when it is unnecessary (e.g., during viral infections in humans), can accelerate the emergence of antibiotic resistance and should be avoided. The gut harbors the largest microbiota in the body, yet other surfaces such as skin, or even the ocular surface (i.e., the cornea and conjunctiva) also contain microbiota. When treating eye diseases, one possible administration route for TMP to avoid systemic administration (and, thus, disruptions to the gut microbiota) is to apply it through eye drops.^11^ However, such a route would also likely disrupt the low-abundance commensal bacteria of the ocular surface^58^ and may predispose to ocular surface infections.^59^ Indeed, studies have shown that repeated exposure to topical antibiotics changes ocular flora, which play an important role in immunologic protection against the proliferation of pathogenic species,^60^, ^61^ and that resistance to TMP is increasing in the microbial flora isolated from ocular infections.^62^ The perturbation of ocular flora homeostasis can result in eye diseases and generate antibiotic-resistant ocular bacteria in the long term.^63^ An ideal strategy for targeting eye-centric DHFR DDs would be to deliver a non-antibiotic compound via eye drops. However, the partition coefficient (logP) of 14a is higher than that of TMP (3.69 versus 1.43), indicating a higher degree of hydrophobicity and potential difficulty in formulating it as an aqueous-based eye drops.

While 14a is a promising and useful lead compound, it is interesting to find that, when it is delivered systemically, it is not as effective in stabilizing DHFR DDs as TMP in the retina. Conversely, it is far more effective than TMP in controlling DHFR DDs in the liver. These observations may be attributed to differences in chemical structures; for example, steric bulk of the dansyl fluorophore may reduce BRB transport, differences in pharmacokinetics and pharmacodynamics in different organs, or a combination of these factors, which needs further investigation beyond the scope of this study. Along this line of thought, aside from validating 14a as a resource for non-antibiotic control of protein abundance, our work implies the possibility of developing a variety of custom-made or rationally identified non-antibiotic compounds that can stabilize DHFR DDs with various pharmacokinetics and pharmacodynamics properties. However, to further apply DHFR DDs for gene therapy, 14a or other compounds need to be more deeply characterized and vetted with regard to their safety and chemical properties in the target organ, especially to be able to replace the current canonical ligand, TMP.

In summary, 14a is a feasible and exciting substitute for TMP to stabilize DHFR DDs *in vivo* without exhibiting antibiotic properties. By replacing TMP with non-antibiotic stabilizers, such as 14a, we believe that the utility of DHFR DDs will evolve from a chemical biology tool to a clinically relevant gene therapy system to treat disease.

## Materials and Methods

### Compounds

TMP was purchased from Sigma-Aldrich (T7883, St. Louis, MO, USA), whereas 12a, 12b, 13a, and 14a were synthesized according to previous studies.^49^ The 14a utilized in this study was 96% pure, as determined by liquid chromatography-mass spectrometry (LC-MS) analysis.

### Mouse Use

All animal experiments followed the guidelines of the Association for Research in Vision and Ophthalmology (ARVO) Statement for the Use of Animals in Ophthalmic and Vision Research and were approved by the Institutional Animal Care and Use Committee (IACUC) of University of Texas (UT) Southwestern Medical Center, Dallas, TX, USA. WT C57BL/6J mice were purchased from the UT Southwestern Mouse Breeding Core and were genotyped to confirm the absence of the potentially confounding *rd8* mutation.^64^ WT BALB/c mice originated from heterozygous breeding schemes from R345W^+/−^ EFEMP1 mice (courtesy of Lihua Marmorstein, private stock at The Jackson Laboratory, Bar Harbor, ME, USA). Equal numbers of age-matched littermate male and female mice were used whenever possible. Mice were provided standard laboratory chow and allowed free access to water, in a climate-controlled room with a 12-h-light/12-h-dark cycle.

### 16S rRNA Sequencing and qPCR of Microbiota

Two groups of six littermate mice aged 8–9 weeks were used for these experiments. TMP or 14a for gavage was prepared by dissolving in 20 μL DMSO and then diluting with 40 μL PEG 400 (Fisher Scientific, Waltham, MA, USA), 4 μL Tween 80 (Fisher Scientific), 20 μL cremaphor (Sigma-Aldrich), and 116 μL 5% dextrose (Fisher Scientific) in nanopure water. Mice were given a single dose equivalent to 1 mg TMP or 3 mg 14a (molar equivalents, since the formula weight of 14a is 3× that of TMP) by oral gavage. Mice fecal samples were collected immediately before oral gavage (day 0) and at days 3 and 7 post-gavage. Two or three pellets of mouse feces were collected and weighed for the qPCR experiment. One separate pellet was saved for 16S rRNA sequencing.

For 16S rRNA sequencing, genomic DNA was extracted using the QIAamp PowerFecal DNA Kit (QIAGEN, Germantown, MD, USA). At least 400 ng DNA of each sample was sent to SeqMatic (Fremont, CA, USA) for 16S V4 sequencing and bioinformatics analysis.

The samples for qPCR were processed to isolate bacterial genomic DNA following a published method.^65^ Specifically, 710 μL of 200 mM NaCl, 200 mM Tris, 20 mM EDTA, and 6% SDS, along with 0.5 mL phenol-chloroform-isoamyl alcohol (pH 7.9) (Ambion, Foster City, CA, USA) and 0.5 mL of 0.1 mm zirconia-silica beads (BioSpec Products, Bartlesville, OK, USA), was added to the tube with feces samples. Samples were then lysed by mechanical disruption with a bead beater (BioSpec Products) for 3 min and centrifuged at 6,800 × *g* for 3 min at room temperature. The aqueous phase was transferred to a Phase Lock Gel tube (5 PRIME, Hamburg, Germany) and spun at 16,000 × *g* for 5 min at room temperature. DNA in the aqueous phase was then transferred to a new 1.5-mL tube and precipitated by adding an equal volume of isopropanol and 1/10 volume of 3 M sodium acetate (pH 5.5) (Ambion). The DNA was then pelleted at 18,000 × g for 20 min at 4°C after incubation at −80°C for 1 h and washed once with 0.5 mL of 100% ethanol. After ethanol was removed and the pellet was air dried, DNA was resuspended in 0.2 mL of 50 mM Tris (pH 8.0), 10 mM EDTA with 20 μg/mL RNase and incubated at 37°C for 30 min. Lastly, DNA extracts were further purified by the PCR Purification Kit (QIAGEN) and quantified by the PicoGreen assay (Life Technologies, Carlsbad, CA, USA). Representative bacteria were amplified and quantitatively analyzed using group-specific 16S rRNA gene primers,^66^ including total bacteria (EUB; forward primer, 5′-ACTCCTACGGGAGGCAGCAGT-3′; reverse primer, 5′-ATTACCGCGGCTGCTGGC-3′), BACT (forward primer, 5′-GGTTCTGAGAGGAGGTCCC-3′; reverse primer, 5′-GCTGCCTCCCGTAGGAGT-3′), LACT (forward primer, 5′-AGCAGTAGGGAATCTTCCA-3′; reverse primer, 5′-CACCGCTACACATGGAG-3′), EREC (forward primer, 5′-ACTCCTACGGGAGGCAGC-3′; reverse primer, 5′-GCTTCTTAGTCAGGTACCGTCAT-3′), CLEPT (forward primer, 5′-GCACAAGCAGTGGAGT-3′; reverse primer, 5′-CTTCCTCCGTTTTGTCAA-3′), and ENTERO (forward primer, 5′-GTGCCAGCMGCCGCGGTAA-3′; reverse primer, 5′-GCCTCAAGGGCACAACCTCCAAG-3′).

### Bacterial Growth Assay

The BW25113 WT DHFR *E. coli* strain was grown overnight in M9 minimal media (supplemented with 0.4% glucose and 0.2% amicase), followed by an optical density 600 (OD_600_) measurement using spectrophotometry. The overnight culture was diluted to 1 × 10^−4^ OD in M9 media and aliquoted into 100-μL volumes in a 96-well plate (Wuxi NEST, Jiangsu, China). TMP and 14a (10 mM in DMSO) were diluted in M9 minimal media at interval concentrations and were combined with culture aliquots at a 1:1 volume ratio (30 μM to 0.005 μM for TMP, 50 μM to 0.033 μM for 14a, final concentration). Plates were placed into a shaker at 37°C, and, using an automated robot system (TECAN, Mannedorf, Switzerland), the bacterial density of each dilution was measured periodically as a function of time. Endpoint data (19–24 h) are presented. Using Prism software (GraphPad, San Diego, CA, USA), growth curves were generated by curve-fitting normalized bacterial densities versus compound concentrations.

### Transient Transfection of 293A Cells

HEK293A cells (70507, Life Technologies) were plated overnight at a density of 100,000 cells per well of a 24-well plate (Corning, Corning, NY, USA). The next day, the cells were transfected with pcDNA DHFR.YFP.HA or pcDNA HA.YFP.DHFR (YFP was tagged with hemagglutinin [HA]) using Lipofectamine 3000 (Life Technologies). Briefly, 100 ng DNA per well was diluted into 250 μL OptiMEM per well (Life Technologies) containing 0.5 μL P3000 reagent per well. This solution was vortexed for 10 s, and 1.5 μL Lipofectamine 3000 was added per well, followed by vortexing (10 s) and a 5-min incubation at room temperature (RT). Next, 1/2 of the original conditioned cell-culture media was removed, and 250 μL of the transfection reagent complex was added to the wells overnight. Cells were then treated with the indicated concentration of TMP or TMP derivative for 24 h, followed by harvesting for western blot using radioimmunoprecipitation assay (RIPA) buffer (Santa Cruz Biotechnology, Dallas, TX, USA) supplemented with protease inhibitors (Halt Protease Inhibitor Cocktail, Fisher Scientific) and benzonase (Sigma-Aldrich). Samples were frozen at −20°C until use.

### ARPE-19 Stable Cell Washout and Viability Experiments

ARPE-19 Tet-ON cells (described previously)^50^, ^67^ expressing a doxycycline-inducible version of DHFR.YFP.HA under the CMV/TO promoter were seeded at a density of either 200,000 cells per well of a 24-well plate (for western blotting experiments) or 50,000 cells per well of a 96-well plate (for viability experiments) and allowed to reach confluency over the course of 2 days. Cells were then induced with doxycycline (100 ng/mL, a concentration that does not affect mitochondria biogenesis)^68^ and the indicated concentration of TMP or 14a for 24 h. Cells used for western blotting were then washed with Hank’s Buffered Salt Solution (HBSS, Sigma Aldrich), incubated with fresh media, and harvested at the indicated time point as described earlier. Samples were frozen at −20°C until use. Cells used for viability experiments were treated with doxycycline and TMP or 14a for 24 h, followed by a resazurin mitochondrial reduction potential assay (described previously,^69^ 30 min, 37°C) and CellTiter-Glo 2.0 assay (10–15 min at RT; Promega, Madison, WI, USA).

### Western Blotting

Cell-culture samples were thawed and spun at 21,000 × *g* for 10 min at 4°C, and supernatants were collected. To prepare mice retina samples, the intravireally injected mice (the procedure is described later) were euthanized by overdose of ketamine-xylazine (180 mg/kg and 24 mg/kg, respectively), and their eyes were enucleated. The anterior segment of the eye was discarded, and the retina was peeled from the posterior eyecup and snap-frozen in liquid nitrogen. The retina samples were homogenized in 100 μL RIPA buffer supplemented with protease inhibitors and benzonase and incubated on ice for 15 min. After centrifugation at 17,000 × *g* for 10 min at 4°C, the supernatant was collected and assayed for protein concentration by a bicinchoninic acid (BCA) assay (Pierce, Rockford, IL, USA). Twenty to 25 μg protein was separated on a 4%–20% Tris-glycine gel, followed by transfer to a nitrocellulose membrane using the iBlot 2 Dry Blotting System (Life Technologies). Protein transfer and uniform loading were verified by Ponceau S, and blots were blocked using Odyssey PBS Blocking Buffer (LI-COR, Lincoln, NE, USA) overnight. Primary antibodies were diluted in 5% BSA in Tris-buffered saline (TBS); anti-HA (1:1,500, clone 2-2.2.14; Pierce, Rockford, IL, USA) and anti-β-actin (1:1,400, 926-42212, LI-COR) were probed for 1 h at RT. Membranes were washed with TBS with 0.5% Tween 20 (TBS-T) followed by incubation with species-specific IRDye-conjugated secondary antibodies diluted in 5% milk (1:15,000, LI-COR) for 40 min at RT. Blots were washed with TBS-T, imaged on LI-COR Odyssey CLx and quantified using Image Studio software (LI-COR).

HEK293T-REx cells stably expressing DHFR.YFP or DHFR.dn-cHSF1 (defined previously)^52^ were treated with either 10 μM TMP or 10 μM 14a for 18 h before harvesting. Cells were lysed in RIPA buffer supplemented with protease inhibitor (Pierce) and 1 mM PMSF. Proteins were separated by a 4%–10% SDS-PAGE gel and then transferred to a nitrocellulose membrane. Following a 30-min blocking step in 5% milk, blots were incubated overnight with the appropriate primary antibody against HSF1 (HPA008888, Sigma-Aldrich) or β-actin (A2228, Sigma-Aldrich) and then for 1 h with the appropriate 680- or 800-nm fluorophore-labeled secondary antibodies from LI-COR Biosciences. Detection was performed on a LI-COR imager.

### qRT-PCR of DNAJB1 and HSPA1A

The relative mRNA expression levels of select heat shock response genes were measured using qRT-PCR. HEK293T-REx cells expressing DHFR.dn-cHSF1 or DHFR.YFP were treated with 10 μM TMP or 10 μM 14a for 18 h prior to challenge with STA-9090 at 500 nM for 6 h. RNA was extracted using the EZNA Total RNA Kit I (Omega, Tarzana, CA, USA). qRT-PCR reactions were performed on cDNA prepared from 1,000 ng total cellular RNA using the High-Capacity cDNA Reverse Transcription Kit (Applied Biosystems, Foster City, CA, USA). The FastStart Universal SYBR Green Master Mix (Roche, Indianapolis, IN, USA) and appropriate primers purchased (Sigma) were used for amplifications (6 min at 95°C, then 45 cycles of 10 s at 95°C, 30 s at 60°C) in a Light Cycler 480 II Real-Time PCR machine. The primers used for DNAJB1 were 5′-TGTGTGGCTGCACAGTGAAC-3′ (forward) and 5′-ACGTTTCTCGGGTGTTTTGG-3′ (reverse); the primers for HSPA1A were 5′-GGAGGCGGAGAAGTACA-3′ (forward) and 5′- GCTGATGATGGGGTTACA-3′ (reverse); and the primers for RPLP2 were 5′-CCATTCAGCTCACTGATAACCTTG-3′ (forward) and 5′-CGTCGCCTCCTACCTGCT-3′ (reverse). Transcripts were normalized to the housekeeping gene RPLP2, and all measurements were performed in technical triplicate and originated from three independent experiments. Data were analyzed using LightCycler 480 Software, v1.5 (Roche), and data are reported as the mean ± 95% confidence intervals.

### Intravitreal Injections

Ten- to 12-week-old C57BL/6J or BALB/c mice were anesthetized with a ketamine-xylazine cocktail (120 mg/kg and 16 mg/kg, respectively), followed by pupillary dilation using cyclopentolate hydrochloride (1%, w/v) and tropicamide (1%, w/v), both from Alcon (Fort Worth, TX, USA). GenTeal eye gel (severe dry eye formula, Alcon) was applied before the procedure to prevent corneal drying. Intravitreal injections were guided by a Stemi 305 stereo microscope (Zeiss, Oberkochen, Germany). The right eye was proptosed by periocular pressure and was pierced by a 30G needle at a 45° angle approximately 1 mm posterior to the supratemporal limbus. The needle tip was directed into the mid-vitreous under direct transparent lens visualization with external illumination. The 30G needle was removed, and a 33G 1/2 needle with a 10° to 12° bevel fitted to a Hamilton micro-syringe (Hamilton, Reno, NV, USA) was inserted into the previous incision at a 45° angle until the needle point was mid-vitreous. Two microliters of rAAV2/2 MAX^70^ encoding for DHFR-YFP 2A mCherry or NanoLuc 2A DHFR-FLuc (7.6 × 10^9^ viral genomes, prepared as described previously^11^ or by the University of North Carolina [UNC] Viral Vector Core, Chapel Hill, NC, USA) was slowly injected into the vitreous over the course of ∼1 min. Following complete injection, the needle was held stable for an additional minute before being slowly removed. For the left eye, the same injection procedure was performed using a sham vehicle (HBSS with 0.14% Tween [HBSS-T]). Post-injection, AK-POLY-BAC antibiotic ointment (Akorn, Lake Forest, IL, USA) and GenTeal eye gel was applied to each eye. Mice were kept warm on a heating pad until regaining consciousness. Forty-eight hours post-injection, eyes were inspected macroscopically, and any mice with discernible ocular deformities (e.g., cloudy eye) were excluded from subsequent studies.

### Intravenous Injection

Ten- to 12-week-old BALB/c mice were injected with 200 μL rAAV2/8 encoding for NanoLuc 2A DHFR-FLuc (5 × 10^10^ viral genomes, prepared by the UNC Viral Vector Core, Chapel Hill, NC, USA) intravenously through tail vein.

### Bioluminescence Imaging

Injected BALB/c mice were placed into the IVIS Spectrum In Vivo Imaging System (UT Southwestern Small Animal Imaging Resource, PerkinElmer, Waltham, MA, USA) under anesthesia by circulating isoflurane. Each mouse was injected intraperitoneally with 150 mg luciferin per kilogram of body weight, and the eyes or the abdomen was imaged for bioluminescence over a 20-min time course with a 1-min interval between every image. The next day, mice were given 1 mg TMP or 3 mg 14a by oral gavage (dissolved as described earlier) and imaged again at 6 h after oral gavage. The total flux number at the peak of the kinetics and the image with the peak number were used for plotting and comparison between “−” and “+” TMP or 14a.

### Human DHFR (hsDHFR) Inhibition Assay

A hsDHFR assay kit (CS0340, Sigma-Aldrich) was used following the manufacturer’s protocol. This assay is based on DHFR’s ability to reduce dihydrofolate (DHF) to tetrahydrofolate (THF) in the presence of NADPH, resulting in a reduction of absorbance at 340 nm. Briefly, a stock solution of hsDHFR (1.5 × 10^−3^ U), DHF (10 mM), and NADPH (10 mM) were dissolved in assay buffer. Methotrexate (2.9 mM), TMP (2.9 mM), and 14a (2.9 mM) were dissolved in DMSO and then further diluted to 10 μM in assay buffer. To prepare the reaction mix, hsDHFR (12.5 μL) was added to 972.5 μL assay buffer. Next, 5 μL potential inhibitor (MTX [50 nM], TMP [50 nM], or 14a [50 nM]) and 6 μL NADPH were added, followed by the addition of 6 μL DHF. Using a PerkinElmer LAMBDA 650 UV/Vis spectrophotometer, reaction progression was recorded with 1-s resolution for 150 s. Initial velocities of the reactions were determined using the molar absorbance difference of 12.3 mM^−1^cm^−1^.

## Author Contributions

H.P. and V.Q.C. performed experiments, analyzed data, and wrote the paper. R.M.S., S.D., and M.R. performed experiments. Y.T.T. and E.T. performed experiments and provided key resources. W.P., M.R.L.S., and M.A.T.B. provided key reagents. A.Y.K. provided key resources. J.D.H. supervised the overall work, performed experiments, analyzed data, and wrote the paper.

## Supplemental Information

Document S1. Figures S1–S4

Document S2. Article plus Supplemental Information
