# Supplementary material for: Non-antibiotic Small-Molecule Regulation of DHFR-Based Destabilizing Domains In Vivo
Source: Mol Ther Methods Clin Dev. 2019 Aug 15;15:27–39. doi: 10.1016/j.omtm.2019.08.002 (PMC6804886; doi:10.1016/j.omtm.2019.08.002)
Supplement: Document S1. Figures S1–S4 [file mmc1.pdf]

OMTM, Volume *15*

Supplemental Information

Non-antibiotic Small-Molecule Regulation of DHFR-Based Destabilizing Domains *In Vivo*

Hui Peng, Viet Q. Chau, Wanida Phetsang, Rebecca M. Sebastian, M. Rhia L. Stone, Shyamtanu Datta, Marian Renwick, Yusuf T. Tamer, Erdal Toprak, Andrew Y. Koh, Mark A.T. Blaskovich, and John D. Hulleman

Supplemental Figure Legends for Peng and Chau et al.

Sup. Fig. 1. Quantitation of representative gut bacteria in mice feces treated with TMP. (A) Eubacteria (EUB, all bacteria), (B) Bacteroides (BACT), (C) Enterobacteriaceae (ENTERO), (D) Eubacterium rectale/Clostridium coccoides group (EREC), (E) Clostridium leptum group (CLEPT) and (F) Lactobacillus/Enterococcus group (LACT) levels determined by qPCR of genomic DNA isolated from mice feces samples collected before (day 0) and after (day 3, 7) TMP oral gavage. Data are represented as mean ± SD of n=6, statistical analysis by Mann-Whitney test compared to the abundance at day 0. * $p<0.05$, ** $p<0.01$, n.s., not significant.

Sup. Fig. 2. Stabilization of DHFR.YFP by TMP-derivatives. (A) Chemical structures of 12a, 12b and 13a. The minimal inhibition concentration (MIC) values of these compounds are greater than 64 mg/mL. (B) Western blot probing DHFR.YFP expression (using the HA tag antibody) at different doses of TMP derivatives or vehicle reagent, DMSO, 24 h after treatment. β-actin is used as internal control. Representative image of three experiments. (C) LI-COR quantitation of DHFR.YFP band intensity relative to DMSO treated sample. Data are represented as mean ± SEM of n=3.

Sup. Fig. 3. Quantitation of representative gut bacteria in mice feces treated with 14a. (A) Eubacteria (EUB, all bacteria), (B) Bacteroides (BACT), (C) Enterobacteriaceae (ENTERO), (D) Eubacterium rectale/Clostridium coccoides group (EREC), (E) Clostridium leptum group (CLEPT) and (F) Lactobacillus/Enterococcus Group (LACT) levels determined by qPCR of genomic DNA isolated from mice feces samples collected before (day 0) and after (day 3, 7) 14a oral gavage. Data are represented as mean ± SD of n=6, statistical analysis by Mann-Whitney test compared to the abundance at day 0. n.s., not significant.

Sup. Fig. 4. Human DHFR inhibition assay. (A) Raw data curves of and *in vitro* human DHFR (hDHFR) activity assay using enzyme treated without inhibitor, with a canonical hDHFR inhibitor, methotrexate (MTX), 14a or TMP (all at 50 nM). (B) Specific activity of hDHFR treated with the indicated compounds. Assays were performed in technical triplicates one to two independent times.

Sup. Fig. 1

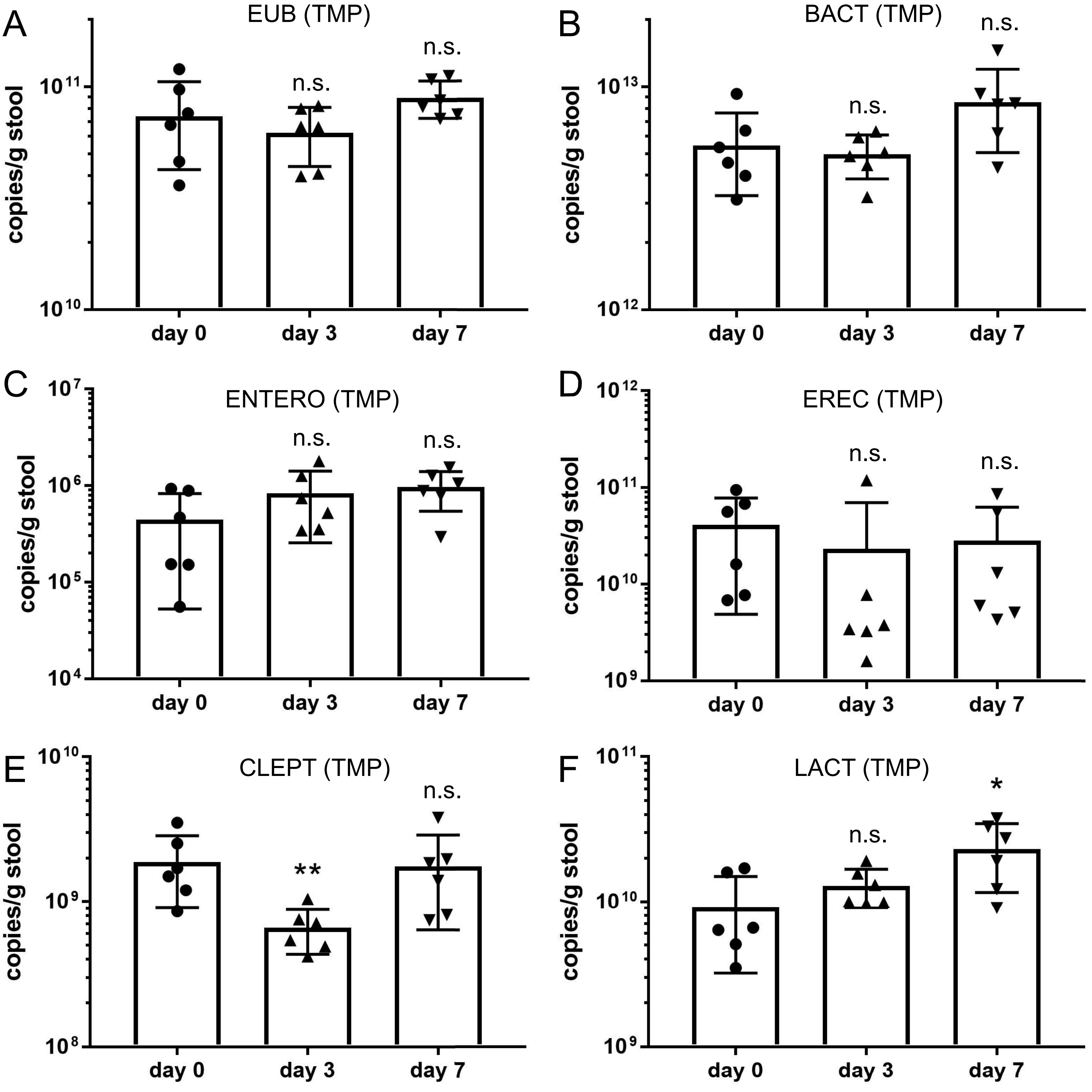
A
EUB (TMP)
B
BACT (TMP)
C
ENTERO (TMP)
D
EREC (TMP)
E
CLEPT (TMP)
F
LACT (TMP)
copies/g stool
day 0
day 3
day 7
n.s.
**
*
10^{10}
10^{11}
10^{12}
10^{13}
10^{4}
10^{5}
10^{6}
10^{7}
10^{8}
10^{9}

Sup. Fig. 2

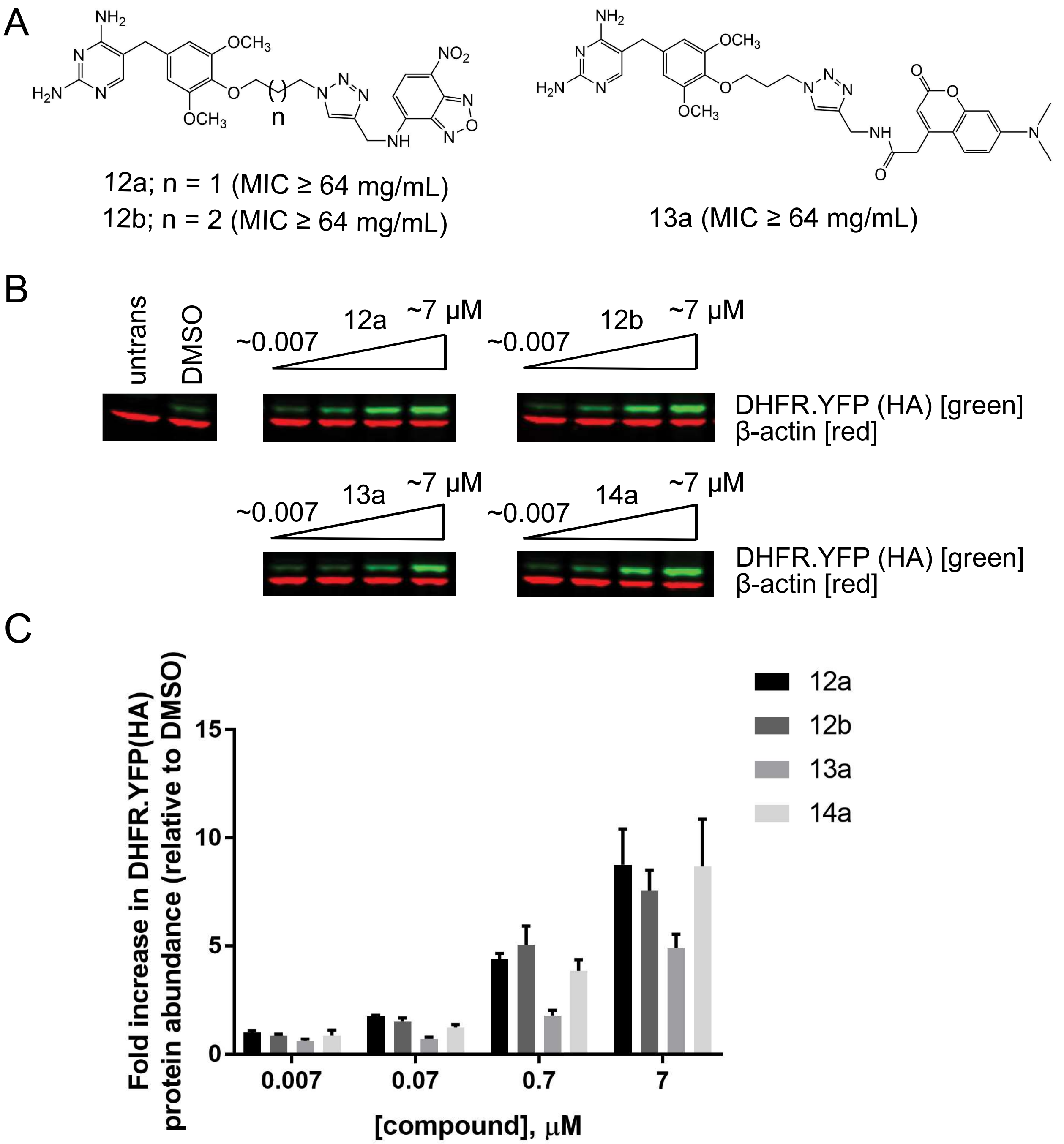
A
12a; n = 1 (MIC ≥ 64 mg/mL)
12b; n = 2 (MIC ≥ 64 mg/mL)
13a (MIC ≥ 64 mg/mL)
B
untrans
DMSO
~0.007
12a
~7 μM
12b
13a
14a
DHFR.YFP (HA) [green]
β-actin [red]
C
12a
12b
13a
14a
Fold increase in DHFR.YFP(HA) protein abundance (relative to DMSO)
[compound], μM
0.007
0.07
0.7
7

Sup. Fig. 3

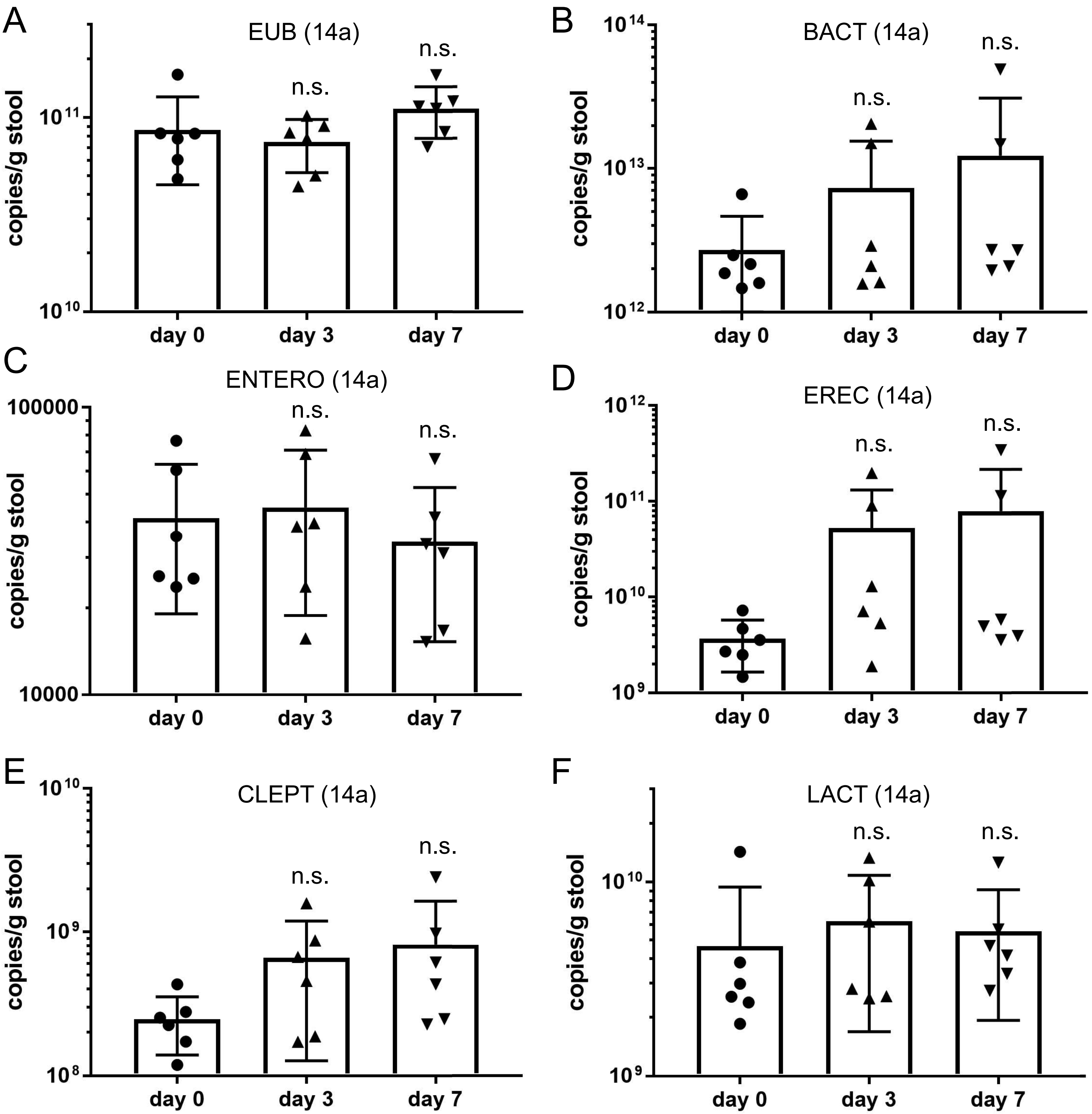
A
EUB (14a)
copies/g stool
10^{11}
10^{10}
n.s.
n.s.
day 0
day 3
day 7
B
BACT (14a)
copies/g stool
10^{14}
10^{13}
10^{12}
n.s.
n.s.
day 0
day 3
day 7
C
ENTERO (14a)
copies/g stool
100000
10000
n.s.
n.s.
day 0
day 3
day 7
D
EREC (14a)
copies/g stool
10^{12}
10^{11}
10^{10}
10^{9}
n.s.
n.s.
day 0
day 3
day 7
E
CLEPT (14a)
copies/g stool
10^{10}
10^{9}
10^{8}
n.s.
n.s.
day 0
day 3
day 7
F
LACT (14a)
copies/g stool
10^{10}
10^{9}
n.s.
n.s.
day 0
day 3
day 7

Sup. Fig. 4

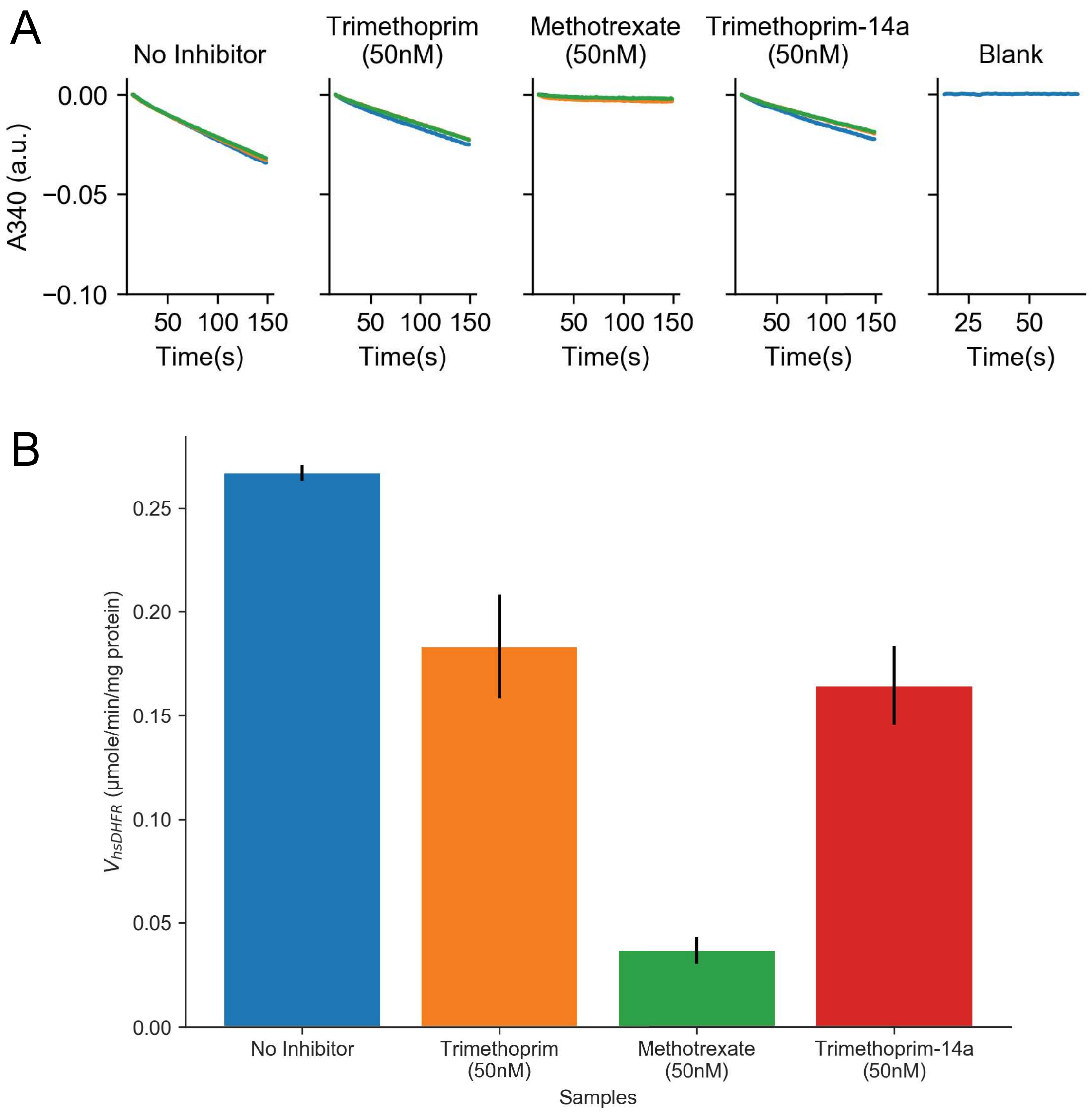
A
No Inhibitor
Trimethoprim (50nM)
Methotrexate (50nM)
Trimethoprim-14a (50nM)
Blank
A340 (a.u.)
0.00
−0.05
−0.10
50 100 150
25 50
Time(s)
B
V_{hsDHFR} (μmole/min/mg protein)
0.25
0.20
0.15
0.10
0.05
0.00
No Inhibitor
Trimethoprim (50nM)
Methotrexate (50nM)
Trimethoprim-14a (50nM)
Samples
